# Endovascular Management of Leriche Syndrome in a 76-Year-Old Woman: A Case Report

**DOI:** 10.7759/cureus.95089

**Published:** 2025-10-21

**Authors:** Ayoub Bellouti, Omar Raisi, Yilmaz Gorur, Anthony Nguyen

**Affiliations:** 1 Cardiovascular Surgery, André Renard Clinic, Liege, BEL; 2 Radiology, André Renard Clinic, Liege, BEL

**Keywords:** aortoiliac occlusive disease, endovascular therapy, infrarenal aortic stenosis, leriche syndrome, primary stenting, tasc d lesion

## Abstract

Aortoiliac occlusive disease (AIOD), or Leriche syndrome, is caused by atherosclerotic stenosis or occlusion of the infrarenal aorta and iliac arteries. It may present with claudication, rest pain, and absent femoral pulses. We report the case of a 76-year-old woman with hypertension and a history of smoking who presented with bilateral lower limb claudication and rest pain. Imaging revealed a high-grade infrarenal aortic stenosis consistent with a TransAtlantic Inter-Society Consensus II (TASC II) type D lesion. She underwent successful endovascular treatment via right femoral access, consisting of percutaneous transluminal angioplasty followed by implantation of an E-Luminexx™ Stent 12×40 mm (Becton Dickinson, New Jersey, USA). Post-operative recovery was uneventful, with improved distal perfusion and normalization of ankle-brachial index values. Follow-up imaging at three months confirmed stent patency. This case illustrates that endovascular management is a safe and effective alternative to open surgery for selected patients with complex AIOD, especially in elderly individuals with comorbidities.

## Introduction

Aortoiliac occlusive disease (AIOD), also known as Leriche syndrome, is a chronic occlusive process caused by atherosclerosis of the infrarenal aorta and iliac arteries, leading to stenosis or occlusion of the vessel lumen. Proximal claudication, sexual dysfunction, and absent femoral pulses constitute the classical triad of Leriche syndrome. Clinically, AIOD can range from asymptomatic disease to severe limb ischemia [1].

Peripheral arterial disease (PAD) is a progressive atherothrombotic condition associated with risk factors, such as smoking, hypertension, diabetes, hyperlipidemia, and physical inactivity. Its prevalence increases with age, with up to 20% of individuals over 65 years affected. Intermittent claudication is the most common symptom, though many patients remain asymptomatic. The ankle-brachial index (ABI) is a reliable diagnostic tool for PAD in both symptomatic and asymptomatic individuals. In addition to ABI, several non-invasive diagnostic methods are available. Duplex arterial Doppler ultrasound provides both anatomical and hemodynamic information, allowing detection and grading of stenoses, as well as assessment of collateral circulation. When further imaging is necessary, CT angiography or conventional angiography helps localize arterial obstructions, assess their length, and evaluate collateral flow and distal vessel patency [2,3].

Traditionally, the management of aortoiliac occlusive disease (AIOD) relied on open surgical techniques, including aortoiliac or aortofemoral bypass grafting and endarterectomy. These approaches have demonstrated excellent long-term patency and are still considered the gold standard in appropriately selected patients. However, they remain associated with significant perioperative morbidity and, in certain cases, non-negligible mortality, particularly among older or comorbid individuals [4].

However, advancements and innovations in endovascular devices have revolutionized the treatment of AIOD, offering a less invasive alternative to open surgery. Endovascular interventions, such as angioplasty, stents, and stent grafts, are particularly valuable for elderly patients or those with significant comorbidities who face high surgical risk. Here we report a 76-year-old woman presenting with AIOD or Leriche syndrome and managed by an endovascular approach using a stent [5].

## Case presentation

A 76-year-old woman patient with a history of Crohn's disease, heavy smoking (15 years, two packs/day, stopped 30 years ago), and long-standing hypertension presented with bilateral claudication over a distance of 50 meters, progressively worsening to intermittent rest pain. Additionally, the patient reported persistent lower limb pain, numbness, and sciatica-like symptoms.

**Figure 1:**
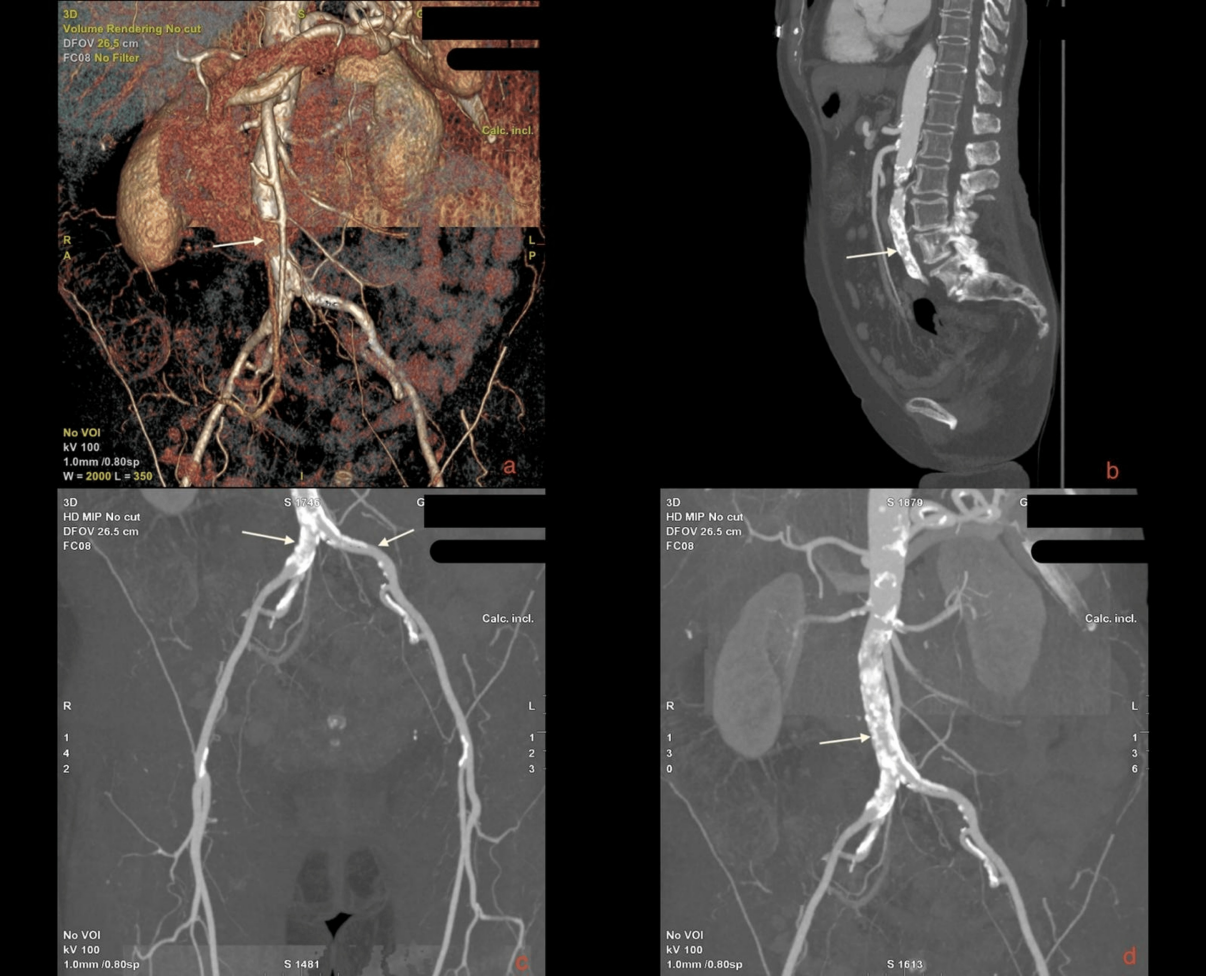
Multiplanar and 3D CT angiographic reconstruction of infrarenal aortoiliac occlusive disease (Leriche syndrome). (a) 3D volume-rendered reconstruction shows a tight infrarenal aortic stenosis with extensive calcifications and collateral vessel development. (b) Sagittal reformatted CT image demonstrates a heavily calcified mixed plaque causing a severe infrarenal aortic stenosis. (c) Coronal maximum intensity projection (MIP) of the iliac and femoral arteries shows reduced perfusion bilaterally with patent distal runoff. (d) MIP image of the abdominal aorta confirms a long-segment calcified stenosis of the infrarenal aorta, consistent with a TASC II type D lesion. Arrows indicate key calcified segments and zones of critical luminal narrowing. TASC II: TransAtlantic Inter-Society Consensus II.

On physical examination, the patient had a blood pressure of 118/52 mmHg, a heart rate of 94 bpm, and a temperature of 36.7°C. Pulses in the femoral arteries were absent, indicating significant vascular compromise. The initial diagnostic imaging included an Angio scanner of the abdominal aorta and lower limb arteries, revealing a stenosis of the infrarenal abdominal aorta of 70-80%, with no significant thrombotic formations. Downstream vascularization of the iliac arteries, femoral arteries, and tibial arteries remained patent but reduced (Figure 1).

Angio scanner results confirmed infrarenal stenosis of the abdominal aorta secondary to mixed atheromatous plaque (mild and calcified). Additional findings indicated normal permeability of the splanchnic arteries and iliac vessels, while the ankle-brachial index (ABI) values were significantly reduced bilaterally. The clinical presentation and imaging findings were consistent with critical limb ischemia due to a significant infrarenal abdominal aortic stenosis.

The patient was diagnosed with AIOD, classified as a TASC II type D lesion, characterized by infrarenal aortic occlusion, commonly referred to as Leriche syndrome. An urgent percutaneous transluminal angioplasty (PTA) was planned as the treatment approach.

The patient underwent an endovascular procedure for the dilatation of the stenosed infrarenal abdominal aorta with the placement of an E-Luminexx™ Stent 12×40 mm (Becton Dickinson, New Jersey, USA).

**Figure 2:**
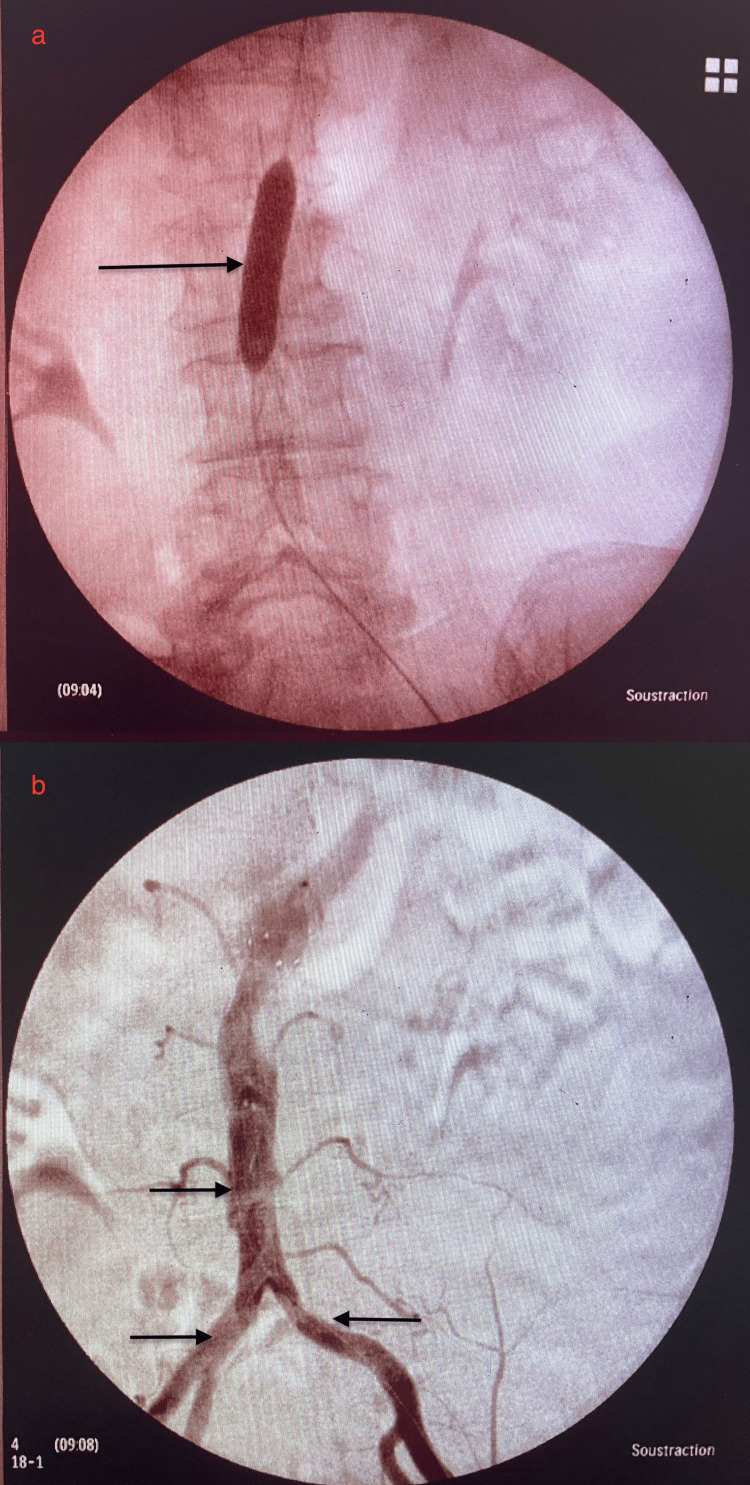
Intra-operative angioplasty of the aorta. (a) Balloon catheter is inflated within the abdominal aorta. (b) Contrast injection shows the aorta and iliac arteries after balloon deflation and removal. The result is satisfactory: the arterial lumen is fully opacified, with visible perfusion into both iliac branches.

After echo-guided percutaneous femoral access via a Radifocus Introducer 6F™ sheath (Terumo, Tokyo, Japan), systemic heparinization with 5000 IU was performed. A Glidewire® hydrophilic coated guidewire (Terumo, Tokyo, Japan) was advanced beyond the lesion, and angiographic sub-occlusion was confirmed. Pre-dilation was performed using a 12×40 mm balloon catheter, followed by the deployment of the stent. Post-dilation was performed to optimize the stent position and ensure complete expansion. Final angiography revealed no residual stenosis, with restored blood flow in the abdominal aorta and femoral arteries. We sealed the femoral artery puncture with the Angio-Seal VIP® vascular closure device (Terumo, Tokyo, Japan) as shown in Figure 2.

Intra-operative findings: Blood pressure measurements showed significant gradients between the suprarenal and infrarenal aorta. The patient was monitored in the post-operative ward with regular assessments of vital signs, limb perfusion, and Doppler studies. There were no signs of hematoma or pseudoaneurysm at the access site. Doppler ultrasound confirmed improved arterial flow to the distal lower limbs. On discharge, the patient demonstrated improved ABI values (right ABI: 0.8; left ABI: 0.77), and no significant complaints were reported.

According to the Rutherford classification, the patient’s condition improved from stage 4 (rest pain) preoperatively to stage 0 postoperatively, reflecting complete resolution of ischemic symptoms and restoration of lower limb perfusion.

The patient was discharged four days after the procedure with instructions to continue antiplatelet bi-therapy (Asaflow 80 mg and Clopidogrel 75 mg) and avoid smoking. Follow-up imaging (CT angiography) at three months demonstrated patency of the stent and improved perfusion to the lower extremities.

## Discussion

The management of aortoiliac occlusive disease (AIOD) has evolved significantly, shifting from an exclusively surgical approach for complex lesions (TASC II C and D) to a more individualized strategy. According to recent European Society of Cardiology (ESC) and European Society for Vascular Surgery (ESVS) guidelines, endovascular treatment is now recommended as the first-line approach for short aortoiliac occlusions (<5 cm). For more extensive or bilateral lesions (TASC II C/D), endovascular therapy may be considered in patients with severe comorbidities, especially when performed by an experienced team and provided that it does not compromise future surgical options [6,7].

Atherosclerotic involvement of the infrarenal aorta manifests in two distinct patterns​. The first pattern consists of focal atheromatous lesions affecting the aortic bifurcation, extending to the distal abdominal aorta and common iliac arteries, with either symmetrical or asymmetrical distribution. The second, less common pattern is characterized by isolated aortic stenosis or occlusion, sparing the aortic bifurcation​. This atypical presentation predominantly affects female and heavy-smoking patients [8].

Compared to open surgery, endovascular treatment offers a less invasive and generally safer alternative. Perioperative mortality is low (typically <5%), and technical success rates are high, around 97%, as demonstrated in studies evaluating techniques such as covered endovascular reconstruction of the aortic bifurcation (CERAB). Major complications are uncommon (approximately 7%), though minor complications can occur in up to 30% of cases. Long-term outcomes are favorable, with a three-year primary patency of approximately 82%, secondary patency of 97%, and reintervention rates around 14%. Most patients achieve durable clinical benefit over time [9].

Endovascular management of aortic occlusive disease primarily involves two techniques: balloon angioplasty alone or primary stenting​​. At our institution, we adopt a primary stenting approach. Device selection is tailored according to anatomical characteristics and lesion morphology. While some authors advocate for direct stent deployment to optimize luminal patency and procedural efficiency, our institution, in line with most studies, opts for pre-dilatation as a preparatory step to enhance procedural success and ensure optimal stent expansion [10,11].

The cost-effectiveness of endovascular intervention is a key consideration in the management of aortic occlusive disease. Although a formal economic analysis was not conducted, several factors suggest the potential financial viability of intraluminal angioplasty, including reduced hospitalization duration, expedited functional recovery, decreased pharmacological requirements, and favorable long-term outcomes. These include reduced hospitalization duration, expedited functional recovery, decreased pharmacological requirements, and favorable long-term outcomes, all of which contribute to its overall therapeutic advantage​​.

## Conclusions

This case highlights the feasibility and efficacy of endovascular treatment as a first-line approach for complex aortoiliac occlusive disease, even in elderly patients with significant comorbidities. The successful management of a TASC II D lesion through percutaneous transluminal angioplasty and primary stenting underscores the evolving role of minimally invasive strategies in cases traditionally reserved for open surgery. With appropriate patient selection, experienced operators, and careful follow-up, endovascular techniques can provide excellent clinical outcomes, improved limb perfusion, and enhanced quality of life while minimizing perioperative risks.
